# Exploring childhood cancer survivor, parent, healthcare and community professionals’ experiences of, and priorities for, using digital health to engage in physical activity: a mixed methods study

**DOI:** 10.1007/s11764-024-01560-z

**Published:** 2024-03-13

**Authors:** Lauren Ha, Suzanne M. Nevin, Claire E. Wakefield, Jacqueline Jacovou, David Mizrahi, Christina Signorelli

**Affiliations:** 1 School of Clinical Medicine, Discipline of Paediatrics and Child Health, UNSW Medicine and Health, UNSW Sydney, Kensington, Australia; 2 Behavioural Sciences Unit, Kids Cancer Centre, Sydney Children’s Hospital, Randwick, Australia; 3 The Daffodil Centre, The University of Sydney, a joint venture with Cancer Council NSW, Sydney, Australia

**Keywords:** Childhood cancer, Survivorship, Physical activity, Focus group, Digital health, Intervention

## Abstract

**Purpose:**

Digital health interventions provide an innovative way to engage childhood cancer survivors in physical activity, yet few studies have explored the priorities of key stakeholders regarding using digital health. We aimed to investigate survivor, parent, and healthcare and community professional (HCP) experiences, priorities, and perceived importance of using digital health to promote physical activity behaviours for survivors.

**Methods:**

Participants rated the importance of digital health features to promote physical activity in a survey. Guided by survey responses, we facilitated online focus groups or semi-structured interviews to discuss participants’ experiences, priorities, and suggestions in-depth. We transcribed the data verbatim and conducted directed content analysis.

**Results:**

Forty participants took part in focus groups or interviews (including 9 childhood cancer survivors aged 8–21 years, 13 parents, and 18 HCP). Four key priorities were identified: health behaviour education, peer and parent involvement, goalsetting, and support from an HCP. There was a strong preference for digital mediums to facilitate physical activity due to its accessibility and convenience. Common intervention suggestions included earlier intervention (before the survivorship period), tailored and age-appropriate programs, a combined diet and exercise approach, and concise education delivery.

**Conclusions:**

This study identified key priorities that may help to promote physical activity behaviours among childhood cancer survivors. Further research is needed to integrate these priorities into health behaviour interventions and evaluate their feasibility and efficacy.

**Implications for Cancer Survivors:**

Incorporating these multi-perspective priorities into future interventions will help to ensure their sustainability, acceptability, and uptake. This will in turn support childhood cancer survivors to develop a healthy lifestyle into adulthood.

**Supplementary Information:**

The online version contains supplementary material available at 10.1007/s11764-024-01560-z.

## Introduction

The 5-year relative survival rate for childhood cancer is approaching 85% in most high-income countries [1–3]. This improved survival rate is largely attributed to treatment advancements and improved supportive care for many cancer types [1, 4]. Despite increasing survival rates, cancer and its related treatments subject young survivors to an increased risk of developing chronic health conditions such as cardiovascular disease [5] and obesity [6]. Evidence shows that engaging in physical activity lowers survivors’ risk of cardiovascular disease [7] and obesity [8] and improves health-related quality of life [9] and fatigue levels [10].

While the importance of engaging in regular physical activity in this population has been recognised, up to 86% of childhood cancer survivors do not meet recommended physical activity guidelines [11, 12], suggesting the need for innovative approaches. Recent interventions have utilised digital health technologies such as telehealth and activity trackers to reach and engage survivors in physical activity [13–16]. Digital health technologies can be useful tools to eliminate barriers to accessing healthcare, especially for rural and remote families living far from well-resourced healthcare sites which are often concentrated in major cities [17]. Using such technologies also aligns well with young people’s preferences for seeking health information and program delivery, with up to 92% of young survivors using the internet to search for information [18, 19]. Whilst most digital health interventions appear to be feasible to deliver and acceptable among survivors and families, the format, delivery, features, and study design of interventions are often heterogenous [14, 20, 21]. Given the diversity of interventions, it is important to investigate the priorities and needs of this growing population to improve physical activity behaviours.

The ‘iBounce’ study was adapted from an existing digital health education program for children without cancer [22]. iBounce was developed to educate young childhood cancer survivors about health behaviours and to promote physical activity using activity trackers and goalsetting. Findings from the iBounce pilot showed that the distance-delivered program was feasible to deliver and acceptable among survivors, yet further research is needed to address participant experiences and perspectives regarding physical activity engagement.

Consumer involvement in the development of health interventions is crucial to understanding the experiences and perspectives of users [23]. However, most interventions developed for childhood cancer survivors do not involve survivors in the design or decision-making process [24]. Research is sparse regarding survivors’ lived experiences and preferences for engaging in digital health physical activity interventions. Additionally, despite parents being key drivers of survivors’ health behaviours, few studies have investigated their preferences of using digital health to encourage a physically active lifestyle for their child [25]. Exploring parent preferences is additionally important given their limited understanding of the benefits of physical activity and their concerns about their child exercising after cancer treatment [26]. Engaging healthcare and community professionals (HCP) such as healthcare providers, researchers, and community members involved in the care of young survivors in the development process of interventions may also be valuable in ensuring the intervention is feasible and implementable in current care pathways. Within Australia, 76% of healthcare organisations with dedicated cancer services offer some type of supportive care or survivorship services for adults or children [27]. These services may involve allied health professionals such as exercise physiologists and physiotherapists [28]. Yet research documenting HCPs’ experiences and preferences within paediatric oncology settings are scarce. Given the limited literature documenting the experiences of survivors, parents, and HCP using different aspects of digital health, this study aimed to identify their experiences and priorities in order to refine the iBounce program. We therefore sought to:

1. Explore survivors’, parents’, and HCPs’ experiences of, and priorities or preferences for, using digital health to promote physical activity behaviours, including their least endorsed features.
2. Explore survivors’, parents’, and HCPs’ suggestions for future digital health/physical activity interventions.

## Methods

### Study design

We used a mixed methods study design to explore the experiences and priorities of survivors, parents, and HCPs. Participants completed a study-specific survey and data from surveys guided online focus group discussions. The number of focus groups and interviews, and the number of participants in each focus group, were guided by a pragmatic approach i.e., participants’ availabilities or preference for a group or private setting. All participants were compensated with a $40 AUD eGift card for their participation. Ethics approval was granted by the Sydney Children’s Hospital Network Human Research Ethics Committee (HREC/18/SCHN/471).

For our qualitative focus groups and semi-structured interviews, we adopted a contextualist approach as we were interested in studying people in the context of their lives [29]. By using contextualism, we allowed our values and practices as paediatric oncology and clinical researchers to shape the collection and analysis of data. This meant that some recruited participants (e.g., clinical researchers, nurses, exercise physiologists) were known to the research team. Rather than attempting to account for or remove our influence as researchers, we combined our experiences to collaborate on discussions. One co-author participated in a focus group discussion as they represented a critical stakeholder with expertise in the area. Their participation was balanced by the representation of other diverse participants (non-authors) offering different viewpoints and balancing perspectives, and they were not involved in the analysis or interpretation of the results. We acknowledge the reflexivity and impact on our data collection and interpretation. Both LH and DM are accredited exercise physiologists and researchers whose work focuses on exercise and paediatric oncology. Authors SN, CW, and CS are psychology researchers with experience in paediatric oncology and qualitative research. JJ was a research assistant at the time of conducting the study. We do not have lived experience of childhood cancer nor experience parenting a child with cancer.

### Participants

We recruited three stakeholder groups: (1) childhood cancer survivors, (2) parents of childhood cancer survivors, and (3) HCPs or relevant stakeholders with paediatric oncology expertise between 2022 and 2023. Participants were deemed eligible if they were (i) aged between 8 and 21 years and diagnosed with childhood cancer and had completed treatment at least 12 months ago or were in the maintenance phase of leukaemia chemotherapy, (ii) a parent of a child or adolescent aged 8–21 years who had completed cancer treatment, (iii) a HCP involved in paediatric oncology or a stakeholder defined as a person involved in an organisation with interest in paediatric oncology, exercise oncology, or digital health. We excluded survivors who were experiencing a medical condition that prohibited them from exercise. Survivors and parents who were previously invited to participate in our pilot intervention for the iBounce study were also invited to participate [22].

We recruited participants through study advertisements posted on social media channels, including X/Twitter and LinkedIn, relevant committees, newsletters, and at an Australian tertiary paediatric hospital, Sydney Children’s Hospital. Previous participants who were invited to our pilot intervention were identified and contacted by the study coordinator via email invitation. If no contact was received after 2 weeks, participants were contacted by the study coordinator via telephone. Potential HCP participants were identified through optional nominations by survivors and families, through study investigators or using the ‘snowballing’ method where participants could suggest other potentially eligible survivors, parents, or healthcare professionals to participate in the study. Participants who opted into the study were sent a study-specific survey and participated in a focus group. Participants who were unable to attend the focus group sessions or who preferred a private interview were offered a semi-structured interview option.

### Data collection

For the quantitative portion of the study, participants were asked to complete an online survey using the survey platform, Qualtrics XM. The survey included participant demographic questions and a single-item question, *please read each of the statements carefully and rate each statement according to how important you think it will be for you/your child/child survivors of cancer.* This list of statements was developed by two researchers (LH, DM) who have expertise in exercise oncology. The lists of statements are related to aspects of digital health and physical activity interventions drawn from our pilot study [22] and other interventions (supplementary material S1). Participants were asked to rate each statement on a 1–9 Likert scale from least (1) to most important (9).

For our qualitative data collection, focus groups were co-facilitated by two researchers at a time (LH, JJ, CS, SN) who had experience in qualitative research, using video-conferencing software (Microsoft Office Teams). The research team conducted 60-min focus groups and 30 to 45-min interviews based on participant availabilities (see supplementary material S2 for the focus group/interview guide). In each focus group, the co-facilitators welcomed the participants and explained the study process. Participants were informed that they were able to withdraw at any time and that they could choose what they felt comfortable sharing in the group. Discussions were guided by the most and least important-rated statements based on what was indicated by participants at the time, participant experiences, suggestions for future intervention designs, and other topics of interest that were in the survey.

### Data analysis

Quantitative data from the surveys were analysed using IBM SPSS Statistics (version 26; IBM Corp) using descriptive statistics to describe participant characteristics and ratings for each intervention feature. We defined ratings of the 9-point Likert scale with 1–3 as ‘least important’, 4–6 as ‘moderately important’, and 7–9 as ‘most important’. To determine the most and least endorsed features between each stakeholder group, we compared the median score for each statement. We used the Kruskal-Wallis test to compare the differences between each stakeholder group.

For the qualitative portion of our study, we recorded and transcribed verbatim in English our focus groups and semi-structured interviews. None of the participants required language translation. The qualitative analytic process began after each focus group and interview was conducted, whereby a coding tree was developed to conceptualise all themes relevant to the research question (i.e., the most and least endorsed features). When no new themes occurred within each stakeholder group, recruitment concluded. Upon completion of data collection, transcripts were analysed using NVivo 12 software (QSR International). The first author (LH) read the transcripts several times and used directed content analysis process involving coding, categorisation, and summarising. Using a deductive approach guided by Elo and Kyngas, a categorisation matrix was developed to code the data according to the most and least endorsed features [30]. Under each feature, the coded comments were grouped into sub-categories based on our pre-determined aims and questions. Comments that did not fit into the categorisation frame were used to create new sub-categories based on the principles of inductive content analysis [30]. A second researcher (SN or JJ) coded 20% of transcripts for each stakeholder group to reduce researcher bias and to ensure inter-rater reliability (survivor transcripts: *k* = 0.67, agreement = 97%; parent transcripts: *k* = 0.77, agreement = 98%; HCP transcripts: *k* = 0.64, agreement = 99%) [31]. To prevent biases in the first author’s interpretations, JJ reviewed the results and conducted peer debriefing to check for the consistency in the themes and key findings [32]. To demonstrate the prevalence of themes and subthemes, we employed a range of quantitative descriptors. This quantifying language was not intended to ‘count’ or ‘tally’ the instances of a theme, but rather to offer insights into the consistencies of themes throughout the data [33]. The term ‘most’ refers to occurrences of the theme within at least 75% of participants’ accounts and ‘many’ refers to 55% to 74% of participants’ accounts. Where the terms, ‘some’ or ‘few’ is used, it refers to occurrences between 20 and 45% [33, 34]. Throughout this study, we followed the consolidated criteria for reporting qualitative research (COREQ) checklist [35].

## Results

A total of 40 participants took part in focus groups or semi-structured interviews (*n* = 9 survivors, *n* = 13 parents, *n* = 18 HCPs) (Table 1). Of the nine survivors who participated, one participant was previously involved in the iBounce study. On average, survivors were aged 14.7 years old (SD 3.2), with 45% identifying as female. Parents were on average 44.8 years old (SD 7.5), 69% female and mostly (84%) white. HCPs were on average 39.9 years old (SD 11.6), 72% female and were 50% physiotherapists or exercise physiologists. Three parents participated in interviews with their child; however, their responses were coded separately. The remaining six survivors opted to participate in semi-structured interviews on their own.

**Table 1:** Participant characteristics

	Survivors (*n* = 9)	Parents (*n* = 13)	Healthcare professionals (*n* = 18)
Age at study (years)^a^, *mean (SD)*	14.7 (3.2)	44.8 (7.5)	39.9 (11.6)
*Range*	8–17	31–53	23–63
Gender, *n (%)*			
Male	4 (45%)	4 (31%)	5 (28%)
Female	4 (45%)	9 (69%)	13 (72%)
Non-binary	1 (10%)	0 (0%)	0 (0%)
Cancer diagnosis^b^, *n (%)*			
Leukaemia	5 (62.5%)	9 (70.0%)	
Neuroblastoma	1 (12.5%)	1 (7.5%)	
Hodgkin lymphoma	1 (12.5%)	1 (7.5%)	
Wilms’ tumour	1 (12.5%)	1 (7.5%)	
CNS tumour	-	1 (7.5%)	
Ethnicity^c^,* n (%)*			
White		10 (84%)	15 (83%)
Aboriginal and Torres Strait Islander		1 (8%)	0 (0%)
Mixed		1 (8%)	2 (11%)
Asian		0 (0%)	1 (6%)
Highest level of education,* n (%)*			
High school		2 (15%)	0 (0%)
Apprenticeship or TAFE certificate/diploma		2 (15%)	0 (0%)
University degree		7 (55%)	4 (22%)
Higher degree (postgraduate qualification)		2 (15%)	14 (78%)
Employment^d^,* n (%)*			
Physiotherapist/exercise physiologist			9 (50%)
Nurse			4 (22%)
Clinical researcher			3 (17%)
Stakeholder			2 (11%)
Direct contact with survivors per week,* n (%)*			
0–1 hours			7 (39%)
1–5 hours			2 (11%)
5–10 hours			2 (11%)
10–20 hours			2 (11%)
20+ hours			5 (28%)

Abbreviations: *CNS* central nervous system, *TAFE* technical and further education

^a^Missing data for survivor age at study (*n* = 2), though we can categorise these two participants as adolescents

^b^Missing data for survivor cancer diagnosis (*n* = 1)

^c^Missing data for parent ethnicity (*n* = 1)

^d^Clinical researchers’ educational background included exercise physiology (*n* = 1) and psychology (*n* = 2). One researcher was also a clinical psychologist. All three clinical researchers conducted research in paediatric oncology. Stakeholder employment included chief executive officer of a paediatric oncology community organisation (*n* = 1) and community project representative (*n* = 1)

### Survey responses

**Fig. 1:**
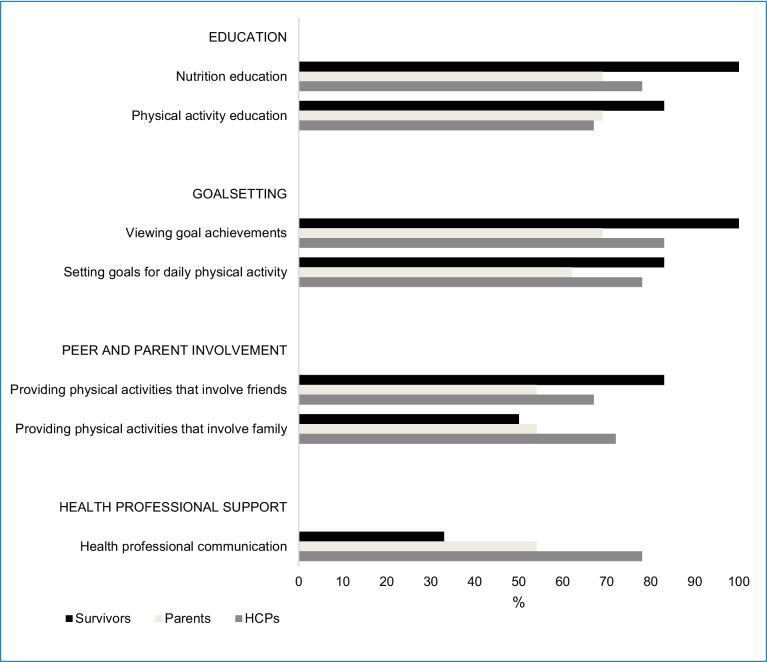
Most endorsed features rated by participants. Abbreviations: HCPs, healthcare stakeholders

**Fig. 2:**
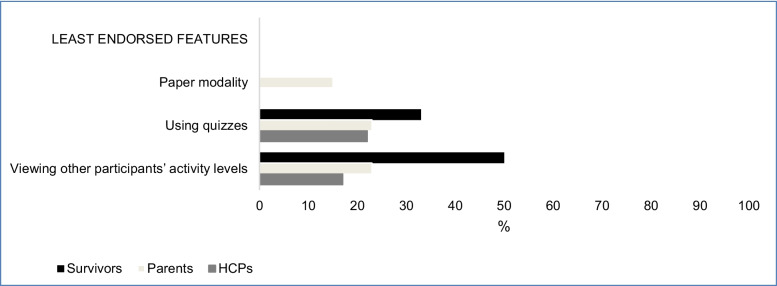
Least endorsed features rated by participants.Abbreviations: HCPs, healthcare professionals

A summary of participants’ top-rated and bottom-rated statements is presented in Figs. 1 and 2, respectively. We did not find any significant differences between stakeholder groups in terms of their preferences, except for the statement, ‘having a social platform with other participants in the program’ (*χ*^2^ = 6.07, *p* < 0.05). Participant ratings for all statements are in Supplementary Material S3.

### Survivors’, parents’, and HCPs’ experiences and priorities

We identified similar priorities for survivors, parents, and HCPs; therefore, their data are grouped under four common themes. Their priorities to promote physical activity for survivors were: (1) health behaviour education, (2) goalsetting, and (3) peer and parent involvement. Parents and HCP additionally identified a fourth theme, (4) health professional support, as a priority. Subthemes within each overarching priority are presented alongside illustrative quotes in Table 2 for survivors and parents and Table 3 for HCP.

**Table 2:** Illustrative quotes for survivor and parent priorities for using digital health to promote physical activity behaviours, and subthemes

Priority	Subtheme	Illustrative quotes
Health behaviour education	Tailored and age-appropriate education relevant to cancer survivors	‘The actual facts behind losing weight and staying healthy and all of that actual scientific stuff. Not just stuff you are seeing on social media because it’s so different from someone that hasn’t gone through trying to lose weight to someone that actually does have past medical issues’ [16-year-old female survivor].
		‘The more we educate him while he’s young on the different physical activities he has to do for his rehabilitation, for his long-term side effects, the more of a voice he’s gonna have as he becomes a teenager [through] to young adulthood’ [mother of 11-year-old survivor].
	Diet and exercise guidance	‘And for you [child’s name], it’s mostly around gaining weight in a healthy way, right? Gaining muscles, becoming more independent’ [mother of 16-year-old survivor].
		‘I guess the big thing for me is actually having instructions and knowing what to do in terms of exercise. I know that when I started a couple of weeks ago [at] the gym, it was very hard to actually know what exercise to do and how to do them correctly’ [16-year-old male survivor].
	Desire for parental educational resources	‘I had no clue what I was in for… I thought kids get extremely fragile and thin… So, I was just like, “eat whatever you want to eat, blah, blah blah. Don’t do any exercise and just chill because you’re sick or whatever.” And really, we should have been keeping up with exercise, still eating healthy’ [mother of 16-year-old survivor].
Goalsetting	Assists with motivation	‘So, with [child’s name], he loses motivation quite quickly unless he has goals and visuals set for him.’ [mother of 11-year-old survivor]
	Setting value-based goals	‘I guess for kids if they’ve got a sport they’re doing, just to set goals…so then they can get back on the field or get back on the court or whatever they do.’ [16-year-old male survivor]
	Collaboration with healthcare professional	‘I think what I find is if I were to set goals myself first and then have someone help me, … because sometimes I might set a really big goal, but I can’t see that because- sometimes I’m not really sure- about the science side or if I’m able to do this physically.’ [16-year-old female survivor]
Peer and parent involvement	Social support	‘If she’s [survivor’s sister] going [to the gym], I want to go. That’s also just kind of a competitive side of me. I’m like, “mm no if you’re going. I’m going. I’m not letting you win!”‘ [16-year-old female survivor]
		‘I think training with a mate or someone from school or your sibling or your mum or your dad- I think that helps a lot. Obviously, you’re training, but you’re also just talking to someone that’s just living a normal life and they’re the people you want to talk to.’ [16-year-old male survivor]
	Involvement is age-dependent	‘I think once they reach a certain age- especially when they’re a teen, they want more independence.’ [mother of 17-year-old survivor]
	Passive support or parent role modelling	‘I think also it’s nice for them [child] to explain a sport to you. If they do a sport that you don’t necessarily understand, I really like listening to the way that they’ve explained it or how the World Cup works or, you know, even just talking about being active makes you wanna do it.’ [mother of 14-year-old survivor].
		‘Maybe when they’re really young, they could be shy or bit hesitant, maybe seeing mum or a dad do it might be helpful.’ [mother of 17-year-old survivor]
Healthcare professional support	Desire for specialist exercise services	‘I think if there were services provided, particularly in that post period after you’ve accessed your intensive physiotherapy and occupational therapy to develop exercise skills, that’s actually going to make a massive difference to the health and well-being of those kids’ long term because it’s really hard to get on a soccer field when you’re 6, when you’ve got no skills. And of course, that has flow[-on] effects for when you’re 14.’ [mother of 13-year-old survivor]
	Challenges with finding exercise support or services	‘[It] was hard just trying to find someone who could help us because we couldn’t really find much through the hospital or any other resources.’ [mother of 16-year-old survivor]
	Cost of exercise services as a barrier	‘They referred her through… a mental health care plan or something. So, you get 5 visits… but it was still quite expensive’. [mother of 16-year-old survivor]
		‘If it’s free, it will be awesome because most of the time- I cannot afford it… It’s always hard, [it’s] difficult for me to have this access to health professionals because I could not afford it.’ [father of 16-year-old survivor]

**Table 3:** Illustrative quotes for healthcare professional preferences for using digital health to promote physical activity behaviours among survivors, and subthemes

Preferences	Subtheme	Illustrative quotes
Health behaviour education	Importance of education to promote healthy habits	‘Especially in children and young adults, I think it’s instilling that kind of knowledge- and healthy habit making at that young age as well.’ [nurse]
	Addresses fears and concerns	‘I know when I see them there’s still a lot of fear about what they can do and so to have that educational component to say “yes it’s OK” or to talk them through those fears is really important.’ [physiotherapist]
	Early and continuing education	‘Cementing it early on too is a really good way to do it.’ [exercise physiologist]
	Individualised education	‘How education is delivered, it’s always to the parents or caregivers. But it depends on the kid and the age and cognition and those kinds of things to how much education they get. But I think that’s what I try to do is also recognise that people are individuals, different people absorb information in different ways and trying to understand and learn from them what’s the best way to give that information across.’ [physiotherapist]
	Need for reliable sources of information	‘As a child who’s gone Googling or a parent of a child who’s gone Googling, and it might be, I think, quite important to get the message about how exercise plays a role because not that long ago it was almost frowned upon in this space.’ [exercise physiologist]
	Challenges faced by HCPs	‘When they’re on treatment and feeling unwell and tired, it’s kind of the last thing on their to-do list*.* So for my personal experience, I don’t think [I] as a nurse was very good at educating as to why exercise is important.’ [nurse]
Healthcare professional support	HCPs can provide tailored and supportive care	‘Ideally you get to a point where they’re able to self-manage and do all those sorts of things. But I think for a lot of these young people, they do need some guidance and direction on how to navigate systems and services and their long-term health.’ [exercise physiologist]
		‘Having a face to face or telehealth interaction would be meaningful because it could make the discussion a lot more individualised. The kids can talk about what’s meaningful to them rather than a handout that’s quite generalised.’ [exercise physiologist]
	Barriers to engage survivors	‘Although they may all access their oncology services in the major hospitals, in the cities, many of them head back to relatively small towns where the capabilities locally are just not available,’ [stakeholder]
		‘I’ve seen that’s very problematic is that in a lot of circumstances, this [physical activity] seems to be spoken about- by other clinicians as an add on. And I think we need to move towards a scenario where it’s talked about as core to their experience when they’re in an oncology setting.’ [stakeholder]
		‘I feel like at the moment I’m never quite sure what resources there are that I can refer patients to’ [clinical researcher]
Goalsetting	Needs to be individualised to the patient	*‘*And your goals need to be more around what they’re interested in or what they’re focused in or being a bit more creative and how you’re [going to] get them moving’ rather than something as simple as step counts, which will work for some kids, but not all.’ [physiotherapist]
	SMART goalsetting format	‘With the patients that I work with, we talk about smart goal setting.’ [exercise physiologist]
	Collaborative approach	*‘*As a health professional, [such as a] community psychologist or exercise physiologist you know what is going to be important for them. But then at the same time for adherence and compliance to some sort of suggestion, it has to come from intrinsic’. [exercise physiologist]
Peer and parent involvement	Support network	‘They provide support to each other in a kind of shared understanding’. [exercise physiologist]
	Fosters positive and healthy relationship with exercise	‘[For] some*,* [exercising] really brings them close together.’ [nurse]
	Identity and normalisation	*‘*The word cancer isn’t all over it, and it’s not just something they’re doing because they have had cancer. Identity wise it could be a really nice way to encourage involvement in things that aren’t so closely attached to cancer.’ [exercise physiologist]
	Dependent on the individual	‘I think comes down to the age. So, for a young child we know that there’s their activity, and- basically their whole lifestyle is fully influenced by their family structure. So, if it’s an active family and if they go to the park, if they do activities together, that child is more likely to take on those behaviours. So, it’s pretty critical to have parents or siblings involved. And then for older adolescence, this sort of becomes less important.’ [exercise physiologist]

Abbreviations: *SMART* smart, measurable, attainable, reliable, timely

#### Health behaviour education

Health behaviour education was perceived as a top priority from survivors and parents. We identified three subthemes: (i) tailored and age-appropriate education relevant to cancer survivors, (ii) diet and exercise guidance, and (iii) desire for parental resources. Survivors revealed a strong desire to learn about physical activity and health and suggested educational topics such as diet and exercise guidance, and tailored lifestyle education relevant to survivors’ history of cancer. Parents agreed that education would provide their child an understanding of why physical activity is important for them, given their cancer history, and how it could be beneficial for them. They also reflected on the benefits of their child learning at a young age so that they can be independent and translate their health behaviours into adulthood, particularly given their increased risk of medical late effects. Some survivors further suggested education for their parents, so that they can better support their child. Similarly, parents revealed the desire for educational resources so that they could better understand the late effects of cancer and its treatments, including education on how to address their child’s physical rehabilitation needs. Some parents reflected that they were unaware at the time of treatment that engaging in physical activity and eating healthy were important for their child’s health.

HCPs also discussed the importance of health behaviour education for young people and families affected by cancer. The subthemes were: (i) the importance of education to promote healthy habits, (ii) addresses fears and concerns, (iii) early and continuing education, (iv) individualised education, (v) need for reliable sources of information, and (vi) challenges faced by HCP. Particularly for young people, early education was perceived as critical to instil knowledge about the benefits of physical activity in improving overall health and wellbeing and to promote habits so that they can continue into adulthood. Education provided to families by HCP was perceived as a top priority, particularly due to the breadth of information available on the internet, which can present a challenge for those families looking for relevant or reliable sources of information. HCPs reflected that many survivors and families read a lot of information related to their diagnosis or treatment and as a result, may have fears or concerns regarding exercise during or after cancer treatment. Whilst HCP discussed the importance of health behaviour education across the cancer continuum, some reflected on the difficulty they had experienced educating young people, especially in the in-patient setting. Reasons included the lack of time in appointments, patients feeling too unwell, concern with overburdening patients, and nurses not feeling well-equipped to provide exercise education or recommendations.

#### Goalsetting

We identified three subthemes among survivors and parents: (i) assists with motivation, (ii) setting value-based goals, and (iii) collaboration with healthcare professional. Survivors and parents perceived goalsetting was important for motivation and tracking progress. Specifically, setting value-based goals to help survivors facilitate their return to sports or prior physical activities were commonly discussed. Survivors spoke of their experiences of creating small goals after cancer treatment to help with regaining their physical fitness and muscular strength. Some used activity trackers to monitor their step counts as a way of motivation. Both survivors and parents suggested goals should be a collaborative effort between the patient and healthcare professional, to ensure that goals are realistic and achievable.

Among HCPs, the subthemes were (i) individualisation, (ii) SMART goalsetting format, and (iii) collaborative approach. HCPs perceived goalsetting as an important feature to encourage behaviour change and intrinsic motivation for survivors. These goals, however, should be individualised and tailored to each patient, depending on their physical capacity needs and personal interests. HCPs also suggested that goalsetting should be a collaborative approach between the clinician and patient, with the patient leading the discussion to ensure goals are meaningful to them and the clinician assisting to ensure goals are achievable. The SMART (specific, measurable, attainable, realistic, timely) approach was prioritised to assist patients in breaking down their goals and ensure they can be achieved.

#### Peer and parent involvement

Survivors and parents prioritised involvement from peers and parents to promote physical activity behaviours. We identified three subthemes among survivors and parents: (i) social support, (ii) passive support or parent role modelling, and (iii) involvement is age dependent. Survivors highlighted the social benefits of exercising with a friend or family member, including making friends from sporting teams and sharing commonalities with family members. They also expressed that exercising with a friend or family member is more enjoyable compared to exercising alone. They shared that exercising with someone they were familiar with would feel more comfortable and motivational and could facilitate friendly competition. Parents also perceived many social benefits of involving peers and parents into future interventions, such as providing motivation to be active and facilitating socialisation. Parents suggested that they can be involved to support their child in both active and passive ways, such as exercising together or listening to their child explain their sport to them. Parents agreed that peer and parent involvement may depend on the age of the child. Those who are younger may benefit from having their parents involved, whilst adolescents may be more influenced by their peers or may prefer to exercise on their own*.*

Among HCPs, the subthemes were: (i) support network, (ii) fosters positive and healthy relationship with exercise, (iii) identity and normalisation, and (iv) dependent on the individual. HCPs recognised that within the hospital setting, parents, carers, and siblings play an important role as a support person and can act as enablers to physical activity. As family members are also affected by a child’s cancer experience, their mutual understanding may reinforce the survivor’s support network and might extend survivors’ intrinsic motivation. Involving peers and parents in physical activity was perceived as a priority to help survivors to foster a positive and healthy relationship with exercise and make the experience more enjoyable and social, rather than a difficult task. Physical activity with peers and parents was also described as a way to normalise the survivors’ cancer experience. HCPs in particular discussed how exercise can be a way to involve survivors in activities that do not relate to cancer.

#### Exercise professional support

We identified three subthemes among parents: (i) desire for specialist exercise services, (ii) challenges with finding exercise support or services, and (iii) cost of exercise services as a barrier. Parents strongly advocated for specialist exercise professional services during their child’s cancer experience, with some highlighting that their exercise physiologist had the greatest positive influence on their child’s health and wellbeing compared to other allied health services. Many parents expressed disappointment that exercise services were not part of routine cancer care and suggested that these services would be highly beneficial for their child’s physical health long-term. Parents reported several challenges with finding exercise support or services from the hospital and the lack of exercise guidance was felt during their child’s treatment period and post-treatment in long-term follow-up care. After the treatment period, they expressed a desire for “normality” and professional direction to adopt an active lifestyle, particularly as they found themselves no longer resembling a “typical” family. The cost of exercise services was also perceived as a barrier. One parent reflected on the unaffordability of allied health services whilst another echoed the expense even after referral to healthcare plans and accessing government rebates.

Among HCPs, the subthemes were (i) health professionals can provide tailored and supportive care, and (ii) barriers to engage survivors. Exercise professionals perceived that they were in an ideal position to provide supportive care for families and to communicate the benefits of physical activity as they are present throughout the cancer trajectory from diagnosis to survivorship. Due to the ongoing complexities that survivors may face, such as their health, potential late effects and navigating the health system, exercise professionals emphasised that their position is to provide support to patients. Discussions about tailoring education were also prominent, with many HCPs recognising the importance of identifying patient barriers and facilitators to exercise.

When asked about their experiences, health professionals noted several barriers to engaging survivors in physical activity. Nurses highlighted the challenges of the in-patient setting for patients to access exercise support due to under-resourced staff and acknowledged that they lack the capacity to follow up with patients once they have been discharged from hospital. Other barriers included limited exercise experts in the field, unavailability of exercise services at the appropriate time, logistical barriers of distance, and the uncertainty of available referrals or resources to provide to families. Health professionals also reflected on how physical activity is often framed as an ‘add on’ component as part of survivors’ rehabilitation, rather as a core component in cancer care.

### Least endorsed features

We identified similar findings for the least endorsed features among survivors, parents, and HCP; therefore, their data are grouped together. The least endorsed features among all participants were: (1) using quizzes to test survivors’ knowledge and (2) using paper as a program modality (Table 4). Parents and HCPs further rated (3) viewing other participants’ activity levels as low priority.

**Table 4:** Illustrative quotes for participants’ least endorsed features to promote physical activity behaviours among survivors

Competitions with other survivors	‘There’s such a variability with the different tumour strains I guess the level of impact that treatment has had on the individual and I guess their initial starting point, I think it’s pretty hard to try and come up with competitions where you’re trying to compare different things.’ [exercise physiologist]
	‘Unless you’re doing it to encourage each other, unless there’s an encouragement component or something. But in terms of turning it into a competition, no.’ [mother of 14-year-old survivor]
	‘For me, I probably wouldn’t have done something like that only because it’d bring back memories and I think- to be training you want to get away from that…’ [16-year-old male survivor]
Quizzes to test survivors’ knowledge	‘Quizzes you can get into the danger of people just pressing the whole way through and not actually doing it. I think it’s important to have education, but…it’s a delicate balance of not overdoing it so people don’t fall off the platform.’ [exercise physiologist]
	‘Sometimes it can kind of feel like it’s a bit of a chore. So, we kind of need to keep the motivation up and keep it fun and really make it distinct from the everyday of, “oh you have to do, schoolwork. You have to do this. You have to do that.”‘ [mother of 11-year-old survivor]
Paper modality	‘Yeah, I just think paper is a little bit old school for younger people.’ [nurse]
	‘I just think with paper and stuff, you can lose it or- I guess if you’re not very good like me, not very organised with that stuff then you can lose it or misplace it and it’s kind of annoying to carry it around with you…Everyone does everything on their phones now, I guess so it’s just easier to have it on there.’ [16-year-old female survivor]

Quizzes were rated as low importance due to their lack of appeal, the possibility of participants not completing them and potentially disengaging from the intervention altogether. Using paper as a program modality to provide education or information to survivors was also perceived as an outdated modality that may be easily lost or not engaging*.* Digital tools were seen as the preferred choice to disseminate information due to convenience, popularity, ease of use and numerous functionalities.

When discussing the prospect of engaging with other peers who have had cancer in an intervention (such as viewing their activity levels), most survivors expressed interest despite our quantitative data showing moderate importance. Some survivors discussed that engaging with other survivors could potentially be a motivating factor or be an opportunity to make new friends. However, others expressed interest only if those other peers were of similar ages. Parents expressed concerns that engagement with other peers may potentially be harmful to their child. Parents and HCPs described that involving other cancer survivors in a physical activity intervention with survivors may be discouraging or disheartening for those who might compare themselves with others who may be achieving more. Participants were concerned that in an online environment, unhelpful comparisons may be exacerbated by not being able to be reassured by a HCP in a face-to-face context. HCPs reflected on the diversity of the cancer population, in terms of cancer diagnoses, treatments, and the individual themselves. Whilst comparisons and challenges with other participants were acknowledged as potentially negative, HCPs agreed that interactions with other peers can also be potentially positive in supporting each other and fostering connection. Parents proposed that if engagement with other survivors was to be included, it should occur in a supportive and facilitated environment where engagement could be monitored and remain optional for survivors. The primary focus of engagement should foster encouragement, rather than competition.

### Intervention design suggestions

We identified four themes when participants described their design suggestions for a future intervention aimed at promoting physical activity: early intervention, tailored and age-appropriate, addressing both diet and exercise, and concise education delivery (Table 5). In terms of timing, parents and HCPs perceived physical activity as a priority for survivors and suggested interventions should be introduced earlier rather than in the post-treatment or survivorship period. Due to the diversity of cancer survivors, both HCPs and parents suggested an optimal intervention should foster autonomy for survivors to access information that is most relevant and tailored to each participants’ unique needs. This information should be age-appropriate, relevant to children and young people with a cancer experience, and reiterated throughout the entire cancer experience. In terms of delivery, participants suggested simple and concise ‘bite-sized’ information including education that is provided in a positive tone, acknowledges diversity and can be optional for survivors to access.

**Table 5:** Illustrative quotes for participant intervention design suggestions

Early intervention	‘I just think stuff needs to happen- from the get-go. Have plans in place from then instead of sort of an afterthought.’ [father of 8-year-old survivor]
	‘I think closer to when I just finished [treatment, it would], probably [be] helpful, but at this stage it doesn’t really affect me as much to need it.’ [16-year-old male survivor]
Tailored and age-appropriate	‘…the age group and individual circumstances. So, I don’t think it’s a one-size-fits-all kind of scenario’ [mother of 14-year-old survivor]
	‘Trying to make a program that’s really individually tailored as much as possible to each person’s unique needs.’ [researcher]
Addresses both diet and exercise	‘We should be designing programs that include both exercise and nutrition.’ [researcher]
	‘I just want to know about what to eat correctly and how to exercise correctly really.’ [16-year-old male survivor]
Concise education delivery	‘A short, sharp, and snappy video would get a lot of people interested. Not something that’s long.’ [12-year-old female survivor].
	‘Videos is a great idea because you can go on to YouTube and… do it on your own in your own lounge room behind closed doors. You don’t have to go out and buy the equipment.’ [mother of 16-year-old survivor].

## Discussion

Our study aimed to explore childhood cancer survivor, parent, and HCP experiences and priorities for using digital health to promote physical activity behaviours among survivors. Survivors, parents, and HCPs identified four key priorities: health behaviour education, peer and parent involvement, goalsetting, and support from a health professional. The least endorsed features were engagement and competition with other participants, using quizzes to test survivors’ knowledge, and using paper resources. Timely, tailored and age-appropriate, intervention designs that address both diet and exercise, and concise education delivery were prioritised.

Survivors, parents, and HCPs identified health behaviour education as an important priority to encourage physical activity behaviours among survivors. This is consistent in previous research with health professionals [36] and young cancer survivors [37] which highlighted the critical role of health professionals in educating survivors to promote the adoption or maintenance of health behaviours, and the desire for young survivors to receive age-appropriate information delivered from health professionals. Parents and survivors in this study also described the need for age-appropriate educational resources, including a focus on both physical activity and diet specific to their child’s needs as cancer survivors. HCPs in this study described multiple barriers to providing health behaviour education for young people affected by cancer, including lack of time, patients feeling too unwell, concern with overburdening patients, and nurses feeling ill-equipped to provide exercise education. The challenges experienced by health professionals in supporting childhood cancer survivors are well documented [38–40], and the potential for nurses to upskill as possible solutions to bridge the gap has been previously been reported [41]. Nurse-led interventions have demonstrated feasibility and acceptability for young people affected by cancer to educate and engage them to improve their physical health and psychosocial outcomes [42, 43].

Peer and parent involvement was perceived as a high priority among survivors, parents, and HCPs. Participants in this study further highlighted that involvement may depend on the age of the child and their personal preferences. One way to potentially promote health behaviour changes for survivors is by involving parents in health behaviour interventions, especially for younger survivors who may be influenced by their parents. Parent involvement in exercise and diet interventions for childhood cancer survivors has demonstrated positive outcomes such as improved eating habits, physical fitness, and reduced fatigue levels [44, 45]. Furthermore, consistent with emerging studies exploring the lifestyle preferences of young people following a cancer diagnosis, this study highlighted involvement from parents and peers without cancer helps to maintain a sense of normalcy [37, 46, 47]. Peer social support can provide important motivators for young people undertaking physical activity, as they can offer emotional support, distraction, and reduce loneliness among children receiving cancer treatment [48, 49]. Previous research has reported on the feasibility and effectiveness of a peer-supported physical activity intervention on cardiorespiratory fitness among children with cancer [50]. Notably, this intervention focused on promoting physical activity through peer encouragement and motivation, rather than fostering a sense of competition. This aligns with the findings of our study, which showed that both parents and HCPs did not endorse competitive elements in an intervention.

Parents in this study expressed a desire for specialist exercise services, yet many described barriers such as finding appropriate exercise support or exercise guidance for their child, or the unaffordability of allied health services. Attending in-person exercise physiology consultations has shown to be highly acceptable among parents of survivors [51]. Mizrahi et al. delivered a single exercise physiology consultation consisting of goalsetting, discussions about physical activity, common barriers, and ways to overcome them, and identifying survivor preferences and needs. Parents reported high satisfaction from the consultation and 95% of parents and survivors recommended the service to other survivors [51]. Future cancer care teams may consider adopting a multidisciplinary approach with the inclusion of an exercise physiologist in the team to provide exercise-specific clinical care for survivors during and after treatment. Whilst some parents acknowledged the expense of allied health services, a potential advantage of telehealth or digital health services may be an alternative solution to provide supportive care for families.

Goalsetting was perceived as a key priority in this study. Among the general population, goalsetting is a commonly used behaviour change technique aimed to increase physical activity [52, 53]. In the childhood cancer population, a recent review found that few physical activity interventions used goal setting as a motivational strategy to monitor and increase daily steps using activity trackers, yet physical activity levels did not increase [54]. HCPs in this study suggested using the ‘SMART’ acronym (smart, measurable, attainable, realistic, timely) to create goals which have been endorsed by leading health organisations such as the American College of Sports Medicine [55]. Yet, empirical evidence has found that SMART goals do not need to be ‘specific’ to be effective whilst ‘challenging’ goals are considered more effective than ‘achievable’ goals [56]. Given many children and adolescents are insufficiently active, spend long periods of time in sedentary activity [12], and rarely improve physical activity levels after participation in interventions [20, 24, 57], alternative goalsetting approaches may be more relevant, such as creating learning goals to facilitate skill acquisition, followed by performance goals to increase effort and persistence [58].

Parents and HCPs in this study suggested the importance of early health behaviour intervention for survivors. Given the evidence that survivors have an increased risk of experiencing excessive weight gain and developing cardiovascular risk factors, both on-treatment and persisting beyond treatment completion, early intervention is appropriate [59]. Unhealthy behaviours such as poor dietary intake and physical inactivity may be more challenging to reverse once children and AYAs become long-term survivors than setting up positive health behaviours early [60]. Consequently, the timing of implementing lifestyle changes may have a critical impact on the success of interventions aiming to improve health outcomes among survivors [61]. A previous study surveyed parents of childhood acute lymphoblastic leukaemia survivors and found that 76% of parents indicated their preference for lifestyle intervention within 3 months of beginning maintenance chemotherapy [62]. Similarly in another study, health professionals also suggested the maintenance phase as an appropriate time to introduce lifestyle interventions due to less intense treatments and adequate time for the young person’s family to adapt to the diagnosis of childhood cancer [62].

The current study contributes to our understanding of the multi-perspective experiences and preferences of using digital health to promote physical activity behaviours among survivors. Our mixed methods study allowed us to investigate salient preferences obtained quantitatively from survivors, parents, health professionals, clinical researchers, and stakeholders, and additionally validate or explore underlying reasons for these preferences through the qualitative data we collected. We recruited a variety of participants, including survivors who had previously participated in our physical activity intervention (the iBounce study), nurses, and relevant clinical researchers with expertise in paediatric oncology, exercise, and behavioural psychology. This is a strength of our study as their specialised knowledge and experiences contribute to the comprehensive understanding of what considerations are important for survivors’ participation in physical activity.

However, our study also had limitations. The way our study-specific survey was framed and possibly how the study was advertised, may have biased participation toward those who are interested in digital mediums and physical activity, and therefore may not be representative of the population. We used the 9-point scale for participants to rate the statements due to its high validity [63] and potential for greater expression of feelings due to the greater number of anchors in the scale [64]. However, we acknowledge one potential limitation is the possibility of participants clustering their ratings toward the higher end of the scale, especially is they found multiple items to be equally important. This could potentially result in less variability in the data and future studies may consider alternative scaling options. Although we were able to identify participants who had previously participated in the iBounce study, we did not ask participants whether they had any experience with other physical activity interventions. While our study provides valuable insights into the preferences of a subset of survivors, the variation in ages, missing age data for two participants, and non-heterogenous and small sample of participants is a limitation and may have an impact on the generalisability of our findings across different age groups of survivors. In the next step of our development process, it is therefore critical to continue the co-design process with survivors and relevant stakeholders to create a meaningful and sustainable program. Fathers were underrepresented in our study, a common issue in psychosocial oncology research [65]. Family members from culturally diverse backgrounds were also underrepresented. We did not ask survivors about their ethnicity, and we did not ask families about their socioeconomic status, including their income and rurality. Including culturally and socioeconomically diverse families in future research will be a key priority in understanding their lived experiences and priorities to support physical activity behaviours. Further research is needed to develop a targeted health behaviour intervention that addresses the needs of both survivors and their families.

## Conclusion

Our study revealed several key priorities to promote physical activity for childhood cancer survivors, obtained from multiple perspectives. Survivors, parents, and HCPs identified that health behaviour education, peer and parent involvement, goalsetting, and support from an HCPs were important priorities to consider for future interventions. These findings will inform the update and development of the ‘iBounce’ intervention for childhood cancer survivors.

## Supplementary Information

Below is the link to the electronic supplementary material.

Supplementary file1 (DOCX 33 KB)

## Data Availability

No datasets were generated or analysed during the current study.
