# Supplementary material for: Exploring childhood cancer survivor, parent, healthcare and community professionals’ experiences of, and priorities for, using digital health to engage in physical activity: a mixed methods study
Source: J Cancer Surviv. 2024 Mar 13;19(4):1403–18. doi: 10.1007/s11764-024-01560-z (PMC12283819; doi:10.1007/s11764-024-01560-z)
Supplement: Supplementary file 1 — Supplementary file1 (DOCX 33 KB) [file 11764_2024_1560_MOESM1_ESM.docx]

Supplementary Materials

Exploring childhood cancer survivor, parent, and healthcare and community professional experiences of, and priorities for, using digital health to engage in physical activity: A mixed methods study.

**Dr Lauren Ha^1,2^, Dr Suzanne Nevin^1,2^, Prof Claire E. Wakefield^1,2^, Ms Jacqueline Jacovou^1,2^, Dr David Mizrahi^3^, Dr Christina Signorelli^1,2^.**

^1^ School of Clinical Medicine, Discipline of Paediatrics and Child Health, UNSW Medicine and Health, UNSW Sydney, Kensington, Australia

^2^ Behavioural Sciences Unit, Kids Cancer Centre, Sydney Children’s Hospital, Randwick, Australia

^3^ The Daffodil Centre, The University of Sydney, a joint venture with cancer Council NSW, Sydney, Australia

**Corresponding Author**

Dr Lauren Ha

[Lauren.ha@unsw.edu.au](mailto:Lauren.ha@unsw.edu.au)

**Supplementary Material S1. List of statements.**

|  | **Statements** |
| --- | --- |
| Goal setting | Being able to set goals for daily physical activity |
|  | Being rewarded if goals are reached |
|  | Being given advice on how to set goals for daily physical activity |
|  | Being able to set their own goals for nutrition |
|  | Being able to view goal achievements |
| Wearable activity trackers | Step counts |
|  | Wearing an activity tracker to record daily physical activity |
|  | Wearing an activity tracker that shows daily physical activity |
| Modality | Using the program on an iPad or tablet device |
|  | Using the program on a phone |
|  | Using the program on a computer or laptop |
|  | Using the program on paper or in a booklet |
| Peer and parent involvement | Providing physical activities that involve friends |
|  | Providing physical activities that involve family |
|  | Doing the program with a friend |
|  | Doing the program with a family member e.g., parents or siblings |
| Cancer peer involvement | Social platform with other participants in the program |
|  | Competitions with other participants in the program |
|  | Viewing other participants’ activity levels |
| Health behaviour education | Learning about physical activity and fitness |
|  | Learning about nutrition |
|  | Learning about cancer |
|  | Using quizzes to test what child has learnt in the program |
|  | Comparing daily activity to physical activity recommended guidelines |
| Health professional communication | Communication platform with a health professional |
| Gamification | Gamifying the program (i.e. making the program like a game). |
| Weight and body composition | Being able to check weight and body composition |

**Supplementary Material S2. Focus Group/Interview Guide**

**Workshop A: childhood cancer survivors – total time: 60-90 min (focus group); 30 min (interview)**

**Sample: maximum 8 participants**

Introduction:

• Welcome participants, introductions and working together

• Remind participants of any guidelines/ground rules of importance for the discussions. This includes highlighting that although their personal experiences provide them with invaluable expertise to inform this discussion, it is completely up to them what and how much they disclose of their own cancer/cancer care experiences. There is no expectation that participants divulge any personal details/experiences that they do not wish to.

• Describe outline of the workshop and the goals of the focus group:

- We will review the top 3-5 statements that participants rated that were most important to them. Discussion.
- We will review bottom 3-5 statements that participants rated that were least important to them. Discussion.

Part 1: Top-rated statements

• Review results of pre-workshop surveys

• Possible discussion questions:

- Why were these most important to you?
- Have you used this in the past? Would you use it again?
- Would you find this helpful for you to increase physical activity levels?

Part 2: Bottom-rated statements / least endorsed

• Review the bottom statements

• Possible discussion questions:

- Why were these least important to you?
- What specifically didn’t work? Why?
- Would there be something else that you would prefer?

Part 3: Other topics of interest

- Can you think of other features that might be important to include for future programs?
- Do you have any other suggestions?

Conclusion:

• Thank participants for their time and summarise the results of the discussions.

• Explain future directions: what we will be doing with the results from the day’s discussions.

**Workshop B: Parents of childhood cancer survivors – total time: 60-90 min (focus group); 30 min (interview)**

**Workshop C: Healthcare and community professionals – total time: 60-90 min (focus group); 30 min (interview)**

**Sample: maximum 8 participants**

Introduction:

• Welcome participants

• Remind participants of any guidelines/ground rules of importance for the discussions. This includes highlighting that although their personal experiences provide them with invaluable expertise to inform this discussion, it is completely up to them what and how much they disclose of their own cancer/cancer care experiences. There is no expectation that participants divulge any personal details/experiences that they do not wish to.

• Describe outline of the workshop and the goals of the focus group:

- We will review the top 3-5 statements that participants rated that were most important to them. Discussion.
- We will review bottom 3-5 statements that participants rated that were least important to them. Discussion.

Part 1: Top-rated statements

• Review results of pre-workshop surveys

• Possible discussion questions:

- Why were these most important to you? Would it be important for your child/young survivors?
- If we were to create a program, would these features be important to include? Why? Any others you can think of?

Part 2: Bottom-rated statements / least endorsed

• Review the bottom statements

• Possible discussion questions:

- Why were these least important to you?
- What specifically didn’t work?
- Would there be something else that you would prefer?

Part 3: Other topics of interest

- Can you think of other features that might be important to include for future programs?
- Do you have any other suggestions?

Conclusion:

• Thank participants for their time and summarise the results of the discussions.

• Explain future directions: what we will be doing with the results from the days discussions.

**Supplementary Material S3. Participant ratings of survey intervention statements.**

| **Statements** | **Survivors**  **(N=6)** | **Parents**  **(N=13)** | **Healthcare and community professionals (N=18)** |  |
| --- | --- | --- | --- | --- |
| Being able to set goals for daily physical activity | 1-3: 0(0%)  4-6: 1(17%)  7-9: 5(83%)  Median (IQR): 7.5 (6.8 – 8.3) | 1-3: 2(15%)  4-6: 4(31%)  7-9: 7(54%)  Median (IQR): 7.0 (5.0 – 7.5) | 1-3: 0(0%)  4-6: 4(22%)  7-9: 14(78%)  Median (IQR): 7.0 (6.8 – 8.0) | χ^2^ (2) = 3.93, p=0.140 |
| Being rewarded if goals are reached | 1-3: 0(0%)  4-6: 3(50%)  7-9: 3(50%)  Median (IQR): 6.5 (5.8 – 8.3) | 1-3: 1(8%)  4-6: 6(46%)  7-9: 6(46%)  Median (IQR): 6.0 (5.0 – 8.0) | 1-3: 1(6%)  4-6: 6(33%)  7-9: 11(61%)  Median (IQR): 7.0 (5.8 – 8.0) | χ^2^ (2) = 0.47, p=0.792 |
| Being given advice on how to set goals for daily physical activity | 1-3: 0(0%)  4-6: 3(50%)  7-9: 3(50%)  Median (IQR): 6.5 (5.8 – 8.3) | 1-3: 1(8%)  4-6: 2(15%)  7-9: 10(77%)  Median (IQR): 7.0 (6.5 – 8.0) | 1-3: 0(0%)  4-6: 2(11%)  7-9: 16(89%)  Median (IQR): 8.0 (7.0 – 9.0) | χ^2^ (2) = 4.30, p=0.116 |
| Being able to set their own goals for nutrition | 1-3: 0(0%)  4-6: 3(50%)  7-9: 3(50%)  Median (IQR): 6.5 (6.0 – 8.3) | 1-3: 0(0%)  4-6: 5(38%)  7-9: 8(62%)  Median (IQR): 8.0 (5.0 – 8.0) | 1-3: 1(6%)  4-6: 4(22%)  7-9: 13(72%)  Median (IQR): 7.0 (5.0 – 8.0) | χ^2^ (2) = 0.13,  p=0.937 |
| Being able to view goal achievements | 1-3: 0(0%)  4-6: 0(0%)  7-9: 6(100%)  Median (IQR): 7.5 (7.0 – 9.0) | 1-3: 1(8%)  4-6: 3(23%)  7-9: 9(69%)  Median (IQR): 8.0 (6.0 – 8.5) | 1-3: 0(0%)  4-6: 3(17%)  7-9: 15(83%)  Median (IQR): 8.0 (7.0 – 9.0) | χ^2^ (2) = 0.25  p=0.883 |
| Step counts | 1-3: 0(0%)  4-6: 4(67%)  7-9: 2(33%)  Median (IQR): 5.0 (4.0 – 7.5) | 1-3: 4(31%)  4-6: 7(54%)  7-9: 2(15%)  Median (IQR): 5.0 (3.0 – 6.0) | 1-3: 3(17%)  4-6: 9(50%)  7-9: 6(33%)  Median (IQR): 6.0 (4.8 – 8.0) | χ^2^ (2) = 2.01,  p=0.366 |
| Wearing an activity tracker to record daily physical activity | 1-3: 0(0%)  4-6: 4(67%)  7-9: 2(33%)  Median (IQR): 6.0 (4.8 – 8.3) | 1-3: 3(23%)  4-6: 7(54%)  7-9: 3(23%)  Median (IQR): 5.0 (3.5 – 6.5) | 1-3: 2(11%)  4-6: 10(56%)  7-9: 6(33%)  Median (IQR): 6.0 (5.0 – 7.0) | χ^2^ (2) = 2.21, p=0.332 |
| Wearing an activity tracker that shows daily physical activity | 1-3: 1(17%)  4-6: 3(50%)  7-9: 2(33%)  Median (IQR): 5.0 (4.3 – 8.3) | 1-3: 3(23%)  4-6: 6(46%)  7-9: 4(31%)  Median (IQR): 5.0 (3.5 – 7.5) | 1-3: 3(17%)  4-6: 7(39%)  7-9: 8(44%)  Median (IQR): 6.0 (5.0 – 8.0) | χ^2^ (2) = 0.99,  p=0.608 |
| Using the program on an iPad or tablet device | 1-3: 2(33%)  4-6: 4(67%)  7-9: 0(0%)  Median (IQR): 4.5 (2.8 – 5.3) | 1-3: 5(38%)  4-6: 4(31%)  7-9: 4(31%)  Median (IQR): 4.0 (2.0 – 7.0) | 1-3: 2(10%)  4-6: 8(45%)  7-9: 8(45%)  Median (IQR): 5.5 (4.0 – 7.0) | χ^2^ (2) = 3.35,  p=0.188 |
| Using the program on a phone | 1-3: 0(0%)  4-6: 5(83%)  7-9: 1(17%)  Median (IQR): 5.5 (4.8 – 6.8) | 1-3: 2(15%)  4-6: 4(31%)  7-9: 7(54%)  Median (IQR): 7.0 (5.5 – 7.5) | 1-3: 1(6%)  4-6: 6(33%)  7-9: 11(61%)  Median (IQR): 7.0 (5.0 – 7.0) | χ^2^ (2) = 1.32,  p=0.518 |
| Using the program on a computer or laptop | 1-3: 2(33%)  4-6: 4(67%)  7-9: 0(0%)  Median (IQR): 4.0 (2.8 – 5.3) | 1-3: 6(46%)  4-6: 5(39%)  7-9: 2(15%)  Median (IQR): 4.0 (2.5 – 5.5) | 1-3: 3(17%)  4-6: 10(55%)  7-9: 5(28%)  Median (IQR): 5.0 (4.0 – 7.0) | χ^2^ (2) = 3.84,  p=0.147 |
| Using the program on paper or in a booklet | 1-3: 2(33%)  4-6: 4(67%)  7-9: 0(0%)  Median (IQR): 4.5 (1.8 – 5.3) | 1-3: 6(46%)  4-6: 5(39%)  7-9: 2(15%)  Median (IQR): 4.0 (2.5 – 5.5) | 1-3: 5(28%)  4-6: 13(72%)  7-9: 0(0%)  Median (IQR): 5.0 (3.0 – 6.0) | χ^2^ (2) = 0.68,  p=0.711 |
| Doing the program with a friend | 1-3: 0(0%)  4-6: 3(50%)  7-9: 3(50%)  Median (IQR): 6.5 (4.0 – 8.3) | 1-3: 1(8%)  4-6: 9(69%)  7-9: 3(23%)  Median (IQR): 6.0 (4.5 – 6.5) | 1-3: 1(6%)  4-6: 9(50%)  7-9: 8(44%)  Median (IQR): 6.0 (5.8 – 8.3) | χ^2^ (2) = 2.84, p=0.242 |
| Doing the program with a family member e.g., parents or siblings | 1-3: 2(33%)  4-6: 0(0%)  7-9: 4(67%)  Median (IQR): 7.0 (2.0 – 9.0) | 1-3: 1(8%)  4-6: 6(46%)  7-9: 6(46%)  Median (IQR): 6.0 (5.0 – 7.0) | 1-3: 0(0%)  4-6: 8(44%)  7-9: 10(56%)  Median (IQR): 7.0 (6.0 – 8.3) | χ^2^ (2) = 1.70, p=0.429 |
| Providing physical activities that involve friends | 1-3: 1(17%)  4-6: 0(0%)  7-9: 5(83%)  Median (IQR): 7.0 (6.0 – 9.0) | 1-3: 0(0%)  4-6: 6(46%)  7-9: 7(54%)  Median (IQR): 7.0 (5.0 – 7.0) | 1-3: 0(0%)  4-6: 6(33%)  7-9: 12(67%)  Median (IQR): 7.5 (5.8 – 9.0) | χ^2^ (2) = 2.77, p=0.251 |
| Providing physical activities that involve family | 1-3: 1(17%)  4-6: 2(33%)  7-9: 3(50%)  Median (IQR): 6.5 (4.5 – 9.0) | 1-3: 2(15%)  4-6: 4(31%)  7-9: 7(54%)  Median (IQR): 7.0 (5.0 – 7.5) | 1-3: 0(0%)  4-6: 5(28%)  7-9: 13(72%)  Median (IQR): 7.5 (6.0 – 9.0) | χ^2^ (2) = 3.37, p=0.185 |
| Social platform with other participants in the program | 1-3: 1(17%)  4-6: 4(66%)  7-9: 1(17%)  Median (IQR): 5.0 (3.5 – 6.0) | 1-3: 2(15%)  4-6: 7(54%)  7-9: 4(31%)  Median (IQR): 6.0 (4.5 – 7.0) | 1-3: 1(5.%)  4-6: 5(28%)  7-9: 12(67%)  Median (IQR): 7.0 (5.8 – 8.0) | χ^2^ (2) = 6.07, p<0.05 |
| Competitions with other participants in the program | 1-3: 1(17%)  4-6: 3(50%)  7-9: 2(33%)  Median (IQR): 4.0 (3.5 – 7.5) | 1-3: 4(31%)  4-6: 5(38%)  7-9: 4(31%)  Median (IQR): 6.0 (3.0 – 7.0) | 1-3: 4(22%)  4-6: 10(56%)  7-9: 44(22%)  Median (IQR): 5.0 (3.8 – 6.3) | χ^2^ (2) = 0.17, p=0.919 |
| Viewing other participants’ activity levels | 1-3: 2(33%)  4-6: 1(17%)  7-9: 3(50%)  Median (IQR): 5.5 (2.8 – 7.5) | 1-3: 6(46%)  4-6: 4(31%)  7-9: 3(23%)  Median (IQR): 4.0 (2.5 – 6.5) | 1-3: 7(39%)  4-6: 8(44%)  7-9: 3(17%)  Median (IQR): 4.5 (2.0 – 5.3) | χ^2^ (2) = 1.36, p=0.507 |
| Learning about physical activity and fitness | 1-3: 0(0%)  4-6: 1(17%)  7-9: 5(83%)  Median (IQR): 7.5 (6.5 – 8.3) | 1-3: 0(0%)  4-6: 4(31%)  7-9: 9(69%)  Median (IQR): 7.0 (6.0 – 8.0) | 1-3: 0(0%)  4-6: 6(33%)  7-9: 12(67%)  Median (IQR): 8.0 (6.0 – 9.0) | χ^2^ (2) = 1.51, p=0.470 |
| Learning about nutrition | 1-3: 0(0%)  4-6: 0(0%)  7-9: 6(100%)  Median (IQR): 7.5 (7.0 – 8.3) | 1-3: 1(8%)  4-6: 3(23%)  7-9: 9(69%)  Median (IQR): 8.0 (5.0 – 8.0) | 1-3: 0(0%)  4-6: 4(22%)  7-9: 14(78%)  Median (IQR): 8.0 (6.8 – 9.0) | χ^2^ (2) = 1.27, p=0.531 |
| Learning about cancer | 1-3: 0(0%)  4-6: 3(50%)  7-9: 3(50%)  Median (IQR): 6.5 (4.8 – 9.0) | 1-3: 3(23%)  4-6: 3(23%)  7-9: 7(54%)  Median (IQR): 7.0 (3.5 – 7.0) | 1-3: 1(5%)  4-6: 5(28%)  7-9: 12(67%)  Median (IQR): 7.0 (5.8 – 9.0) | χ^2^ (2) = 3.74, p=0.154 |
| Using quizzes to test what child has learnt in the program | 1-3: 1(17%)  4-6: 3(50%)  7-9: 2(33%)  Median (IQR): 5.5 (4.3 – 7.5) | 1-3: 3(23%)  4-6: 7(54%)  7-9: 3(23%)  Median (IQR): 5.0 (3.5 – 6.5) | 1-3: 5(28%)  4-6: 9(50%)  7-9: 4(22%)  Median (IQR): 5.0 (3.0 – 6.3) | χ^2^ (2) = 0.66, p=0.718 |
| Comparing daily activity to physical activity recommended guidelines | 1-3: 0(0%)  4-6: 2(33%)  7-9: 4(67%)  Median (IQR): 7.5 (6.0 – 8.3) | 1-3: 1(8%)  4-6: 9(69%)  7-9: 3(23%)  Median (IQR): 5.0 (4.0 – 6.5) | 1-3: 4(22%)  4-6: 5(28%)  7-9: 9(50%)  Median (IQR): 6.5 (3.8 – 7.0) | χ^2^ (2) = 5.23, p=0.073 |
| Communication platform with a health professional | 1-3: 0(0%)  4-6: 4(67%)  7-9: 2(33%)  Median (IQR): 6.0 (4.8 – 7.5) | 1-3: 1(8%)  4-6: 5(38%)  7-9: 7(54%)  Median (IQR): 7.0 (5.5 – 7.0) | 1-3: 0(0%)  4-6: 4(22%)  7-9: 14(78%)  Median (IQR): 7.0 (6.8 – 8.0) | χ^2^ (2) = 4.48, p=0.106 |
| Gamifying the program (i.e. making the program like a game). | 1-3: 1(17%)  4-6: 3(50%)  7-9: 2(33%)  Median (IQR): 6.0 (3.8 – 7.5) | 1-3: 1(8%)  4-6: 5(38%)  7-9: 7(54%)  Median (IQR): 7.0 (4.5 – 7.0) | 1-3: 5(6%)  4-6: 4(22%)  7-9: 13(72%)  Median (IQR): 7.0 (6.0 – 8.0) |  |
| Being able to check weight and body composition | 1-3: 0(0%)  4-6: 2(33%)  7-9: 4(67%)  Median (IQR): 7.0 (5.5 – 7.5) | 1-3: 3(23%)  4-6: 6(46%)  7-9: 4(31%)  Median (IQR): 5.0 (4.0 – 7.5) | 1-3: 2(11%)  4-6: 11(61%)  7-9: 5(28%)  Median (IQR): 5.5 (4.8 – 7.0) | χ^2^ (2) = 2.09,  p=0.351 |

*Scores 1-3=Not important; 4-6=Moderately important; 7-9=Very important*
